# Efficacy of a cluster randomized controlled parental HIV disclosure intervention trial among parents living with HIV in China: evaluation based on the health action process approach

**DOI:** 10.3389/frph.2025.1499481

**Published:** 2025-05-09

**Authors:** Xiaoming Li, Ran Zhang, Wendi Da, Yuejiao Zhou, Zhiyong Shen, Shan Qiao

**Affiliations:** ^1^ Department of Health Promotion, Education, and Behavior, Arnold School of Public Health, University of South Carolina, Columbia, SC, United States; ^2^ Arnold School of Public Health, South Carolina SmartState Center for Healthcare Quality, University of South Carolina, Columbia, SC, United States; ^3^ Guangxi Zhuang Autonomous Region Center for Disease Prevention and Control, Nanning, Guangxi, China

**Keywords:** parents living with HIV, parental HIV disclosure, health action process approach, intervention efficacy, randomized controlled trial

## Abstract

**Introduction:**

Parents living with HIV (PLH) face psychosocial challenges, including disclosing their HIV status to their children. Parental HIV disclosure is critical for reducing stigma, improving psychosocial well-being, and enhancing family cohesion. This study employed the Health Action Process Approach (HAPA) to assess the efficacy of a theory-based intervention aimed at facilitating HIV disclosure among PLH with children aged 6-15 in Guangxi, China.

**Method:**

Data from a randomized controlled trial involving 791 PLH were analyzed using a multigroup first-order manifest Markov Chain model to investigate transitions through the pre-intention, intention, and action stages over two follow-up periods (6 and 12 months).

**Results:**

The intervention significantly facilitated progression from pre-intention to action (OR = 3.43; 95% CI: 1.17, 10.01) but did not affect the transition from pre-intention to intention (OR = 1.02; 95% CI: 0.47, 2.20) or influence movement within the intention stage.

**Discussion:**

These findings suggest the need for stage-specific interventions to enhance disclosure practices. Future research should focus on identifying psychosocial predictors of disclosure and adapt interventions to the distinct stages of the disclosure decision-making process.

## 1 Introduction

As of 2023, 39.9 million people were living with HIV worldwide, with 30.7 million receiving antiretroviral therapy (ART) (1). In China, there were 1.26 million people living with HIV, with the improvements in ART efficacy and reduced side effects (2). Advancements in ART have transformed HIV into a manageable chronic condition, allowing people living with HIV to live longer, healthier lives. Consequently, many parents living with HIV (PLH) are raising their children into adolescence and beyond. As children mature, parents may increasingly consider disclosing their HIV status, believing that older children possess greater cognitive and emotional capacity to understand the implications of HIV. However, this decision presents significant challenges, including determining the optimal timing, appropriate content, and manner of disclosure. Parents must navigate a delicate balance between honesty and protection, fearing potential stigma or emotional distress. Parental HIV disclosure remain a complex and often distressing process for many PLH.

Despite international guidelines emphasizing the importance of HIV disclosure, disclosure rates remain low in China. According to the China Stigma Index Report, less than half of PLH reported that their children were aware of their parents' HIV status (3). The World Health Organization (WHO) recommends parental HIV disclosure for school-aged children, citing mutual benefits for both caregivers and children, including improved emotional support, better understanding of health conditions, and greater adherence to treatment regimens (4). However, disclosure is often fraught with fear, including concerns about stigma, secondary disclosure by children, and potential negative emotional responses (5). A 2012 survey revealed that only 25% of Chinese parents with children aged 5–16 had disclosed their HIV status to their children, showing the hesitance among PLH to engage in this process (6).

The process of HIV disclosure is complex and involves multiple stages, requiring careful planning and preparation, particularly when disclosing to children. Since children's cognitive and emotional capacities vary by developmental stage, disclosure requires tailored strategies that take these differences into account. The Disclosure Decision-Making Model (DDMM) and the Disclosure Process Model (DPM) were used to guide the development of the intervention, providing a structured foundation for decision-making and communication strategies in parental HIV disclosure (7, 8). Studies have shown that unplanned or forced disclosures, often driven by external pressures, tend to result in poorer psychosocial outcomes for both the parent and the child (9). However, a significant gap exists in intervention evaluation studies regarding the consideration of HIV disclosure as a staged process. A systematic review identified four intervention studies aimed at promoting parental HIV disclosure, but all treated disclosure as a single event, rather than a multi-state process (10). For example, two studies measured HIV disclosure action as a binary outcome (yes-or-no event), while other used simple categorical scales to assess whether disclosure had occurred (10). These approaches fail to capture the complexity of the disclosure process, which includes intention formation, preparation, and gradual disclosure, often spread over time.

To address this gap in intervention evaluation, the Health Action Process Approach (HAPA) was adopted as the theoretical framework for this intervention evaluation due to its explicit focus on stage transitions and its well-established application in health behavior change interventions. HAPA's emphasis on self-efficacy, intention formation, and action planning allowed for a comprehensive evaluation of how participants progressed through the disclosure process over time (11). HAPA posits that individuals move through an ordered set of qualitatively distinct stages (i.e., pre-intention, intention, and action), each requiring tailored strategies to facilitate progression (11). This distinction is particularly relevant to parental HIV disclosure, where PLH may remain in a particular stage due to psychological, social, or structural barriers, necessitating specific interventions to facilitate progression. In the context of parental HIV disclosure, HAPA suggests that PLH progress through three key stages: (1) the pre-intention stage, in which people have not yet decided to act; (2) the intention stage, in which people have decided to act but have not yet started action; (3) the action stage, in which individuals make actual behavior change (12).

Given the central role that stage transitions play in models like HAPA, this study applies the HAPA framework to access the efficacy of a theory-based intervention aimed at facilitating parental HIV disclosure among PLH in Guangxi, China. Specifically, this study examines where the intervention influences stage transitions between pre-intention, intention, and action, and whether it affects the overall pattern of forward and backward transitions. By focusing on stage transitions, this study aims to provide more comprehensive understanding of the HIV disclosure process and offer insights into how interventions can be optimized to support PLH at different stages of disclosure. The primary research questions are: (1) Does the intervention affect specific stage transition probabilities? (2) Does the intervention affect the overall pattern of HIV disclosure stage transition, including forward or backward transitions?

## 2 Methods

### 2.1 Study setting and participant recruitment

The parental HIV disclosure intervention, known as *Interactive Communication with Openness, Passion, and Empowerment* (iCOPE), was implemented in Guangxi Zhuang Autonomous Region (“Guangxi”), a southwestern province of China with the third-highest HIV prevalence (13, 14). The iCOPE intervention was designed as a randomized controlled trial (RCT) aimed at helping PLH disclose their HIV status to their uninfected children in a culturally and developmentally appropriate manner. The intervention targeted both PLH and health care providers, focusing on improving disclosure communication skills and fostering psychosocial support (13). The cluster RCT, conducted from 2012 to 2018, evaluated the efficacy of intervention by comparing outcomes between an intervention group and a control group (15).

To ensure comprehensive coverage, the study selected the top two cities (urban centers) and the top eight rural counties with the highest reported HIV/AIDS cases in Guangxi as study sites. From these areas, 40 clinics were randomly selected with at least 200 HIV/AIDS cases. Within each clinic, 20 PLH who had not yet disclosed their HIV infection to their seronegative children aged 6 to 15 years were randomly recruited. Cluster randomization was applied to assign each clinic to either the intervention or control group.

#### 2.1.1 Randomization and allocation concealment

Clinics were randomly assigned to either the intervention or control group using a randomization sequence. Assignment was concealed from participants and facilitators until after baseline data collection to prevent bias.

#### 2.1.2 Blinding

Survey interviewers (i.e., CDC staff) were blinded to the intervention assignment, ensuring that data collection was unaffected by group allocation. However, facilitators (i.e., health educators from the provincial CDC) were not blinded due to the nature of the intervention, as they were directly involved in the delivery of the intervention or control conditions.

To be eligible for the iCOPE intervention, PLH needed to meet the following criteria: (1) be at least 18 years of age, (2) have a confirmed diagnosis of HIV or AIDS, (3) reside with at least one child aged 6–15 years, and (4) have not disclosed their HIV status to their child. Both biological and non-biological parents, if they were the legal and primary guardians of the child, were eligible to participate, although the number of non-biological parents was small. Exclusion criteria included (1) having linguistic, cognitive, or physical impairments that could hinder participation, (2) being currently incarcerated or institutionalized due to drug use or commercial sex work, and (3) having plans to permanently relocate outside the province within a year. Potential participants were referred by medical staff or case managers at the 40 selected clinics (16). Local team members screened individuals for eligibility and provided detailed information about the study's design, potential risks and benefits, and confidentiality protections.

### 2.2 Description of intervention

#### 2.2.1 Intervention condition

The intervention comprised five interactive training sessions, each lasting two hours. The sessions focused on three core components. First, parents were introduced to the stages of childhood cognitive development in relation to parental illness, emphasizing the concept of a child's readiness for disclosure. Second, the intervention aimed to enhance parents' cognitive and behavioral skills concerning HIV disclosure. Specific topics included understanding the benefits and risks of disclosure, practical guidance on how and what to disclose, and recognizing disclosure as an ongoing, evolving process. Third, the sessions provided support to improve parents' psychosocial well-being, helping them adapt to living with HIV/AIDS through discussions and strategies focused on coping with their infection or illness.

#### 2.2.2 Control condition

The control group participated in a series of five interactive training sessions on nutrition education, with each session lasting two hours. The sessions were designed to address three key areas: enhancing parents' knowledge of nutrition, including the importance of food variety and proper nutrition for a growing child; promoting healthy diet and cooking practices, such as managing fat, salt, sugar intake, and the inclusion of fruits, vegetables, and minerals; and ensuring food safety. The decision to use nutrition education as the control condition was informed by community needs and stakeholder input. While all participants living with HIV receive routine HIV care through government-sponsored programs, many lack access to supplementary resources such as nutrition education. Furthermore, selecting nutrition education as an active and attention-matched control condition was intended to minimize placebo or expectancy effects, as participants in both groups received an intervention that required active engagement.

At each participating clinic, a minimum of two health care providers were recruited and trained to serve as facilitators for the intervention. These facilitators were responsible for administering either the parental HIV disclosure program or the nutrition education program, depending on the condition allocated to their clinic. Both the intervention and control sessions were conducted once a week over five weeks at the clinics.

### 2.3 Data collection

Data were collected through a baseline survey and six follow-up surveys conducted every six months (13). Trained survey interviewers (CDC staff) administered surveys to the parents individually in private rooms, such as doctors' offices, at the district or township clinics. Each question in the survey was read aloud by the survey interviewer, and participants provided verbal responses. Clarifications were provided when needed. Written informed consent was obtained from all participants prior to participation in the study. The study was approved by the Institutional Review Boards of Wayne State University, University of South Carolina, and Guangxi Center for Disease Control and Prevention (CDC).

#### 2.3.1 Sample size and power analysis

The sample size estimation was based on a power analysis accounting for the clustered design of the study. Given the lack of empirical data on the effects of parental disclosure interventions in China or other low- and middle-income countries, we conservatively assumed a smaller-than-medium effect size (Cohen's d = 0.35) for the long-term intervention effect. The initial sample size was 791 participants at baseline, with 690 remaining at the 36-month follow-up. Since the unit of randomization was the clinic rather than individual participants, the sample size calculation was adjusted for clustering effects using an intraclass correlation coefficient of 0.10, following standard procedures for determining an effective sample size in cluster-randomized trials. After this adjustment, the effective sample size was 363. According to power calculations, this sample size provided 91% power to detect an effect size of 0.35 at a significance level of α = 0.05, ensuring adequate statistical power to assess the intervention's efficacy.

### 2.4 Measures

#### 2.4.1 HIV disclosure stage

To assess the stage of HIV disclosure, a single question was asked during each follow-up period. Participants were asked to select their current disclosure status based on the following six categories: 1 = “No disclosure in the past six months and no intention to start”, 2 = “No disclosure in the past six months but intends to start”, 3 = “No disclosure in the past six months but has made a plan”, 4 = “Started disclosing but has not mentioned HIV”, 5 = “Started disclosing with the mention of HIV”, and 6 = “Started disclosing, and including mentioning HIV and how I was infected”. These categories were then collapsed into three broader stages based on the HAPA (12). Participants were classified as pre-intenders (response 1), intenders (responses 2–3), or actors (responses 4–6). These three disclosure stages were used for further statistical analysis (12).

#### 2.4.2 Baseline characteristics

Baseline characteristics were collected at the parent and child levels. Parent-level variables included socio-demographic and HIV-related factors. Socio-demographic data included age, gender, marital status, and level of education. HIV-related variables captured included route of HIV infection, time since diagnosis, ART uptake, CD4 count (used as an indicator of immune function), and viral load (the concentration of HIV in the bloodstream). Child-level variables included the child's gender and age. Age groups were categorized into three brackets: 6–9 years, 10–12 years, and 13–15 years.

### 2.5 Analysis

**Figure 1:**
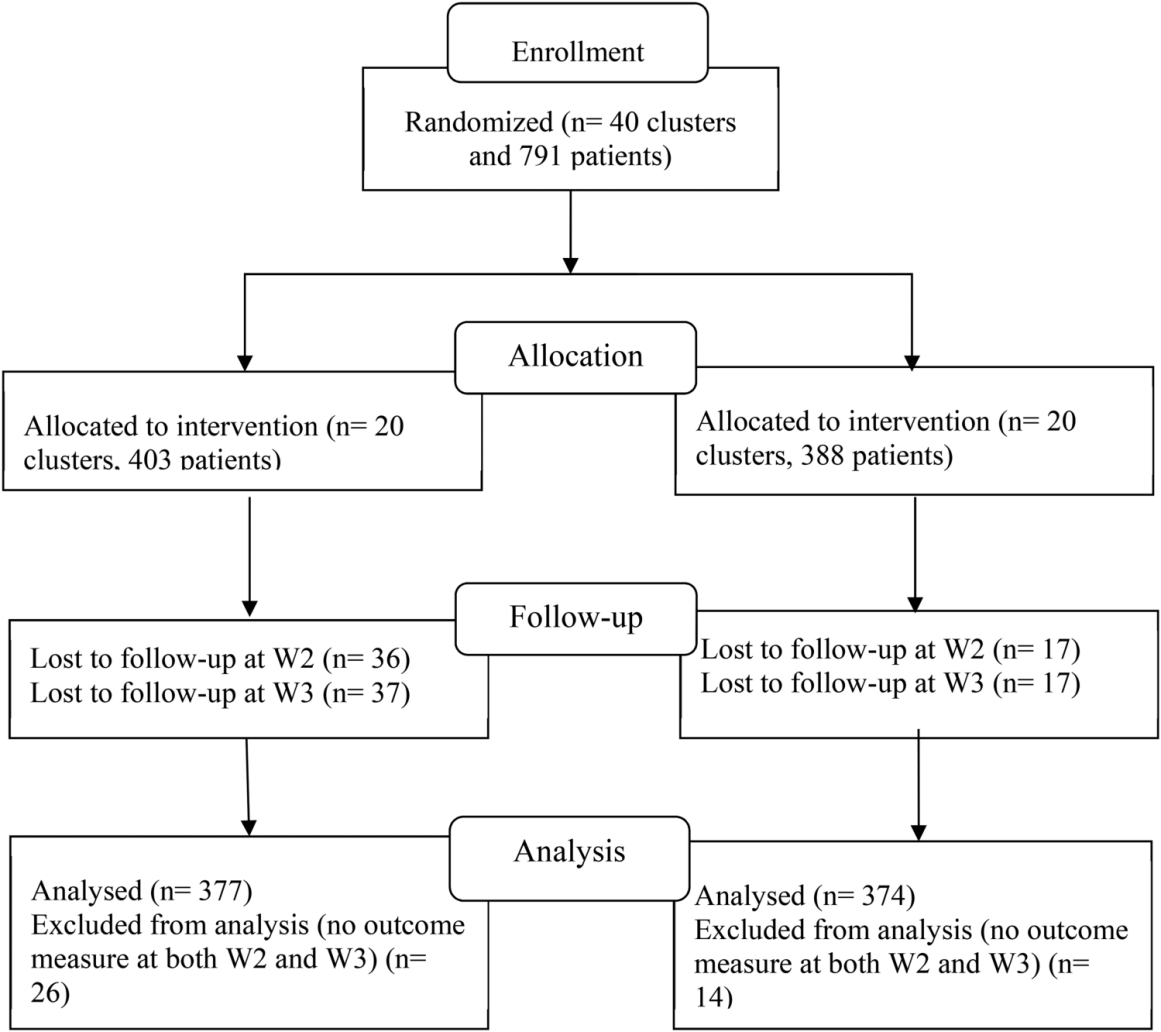
CONSORT flow diagram of 791 participants.

Baseline characteristics (W1) and HIV disclosure stages from the first two follow-up assessments (W2 at 6 months and W3 at 12 months) were utilized for this analysis. A total of 791 participants completed the baseline survey. However, participants who did not respond to the HIV disclosure question at both W2 and W3 were excluded. This resulted in a final sample of 374 participants from the intervention group and 377 from the control group, as depicted in the CONSORT flow diagram (Figure 1).

Data analysis was performed using Stata 13.0 (17) and Mplus 7.4 (18). Prior to the main analysis, a randomization check and attrition analysis were conducted. The distribution of HIV disclosure stages by intervention group was compared using Somers' D test.

#### 2.5.1 Markov chain model of disclosure transitions

The transition between HIV disclosure stages was modeled using a first-order manifest Markov Chain. The model assumed two conditions: (1) the stage occupied at W3 was dependent solely on the stage at W2 (first-order assumption), and (2) no measurement error occurred in the disclosure stages (manifest assumption). This approach involved two multinomial logistic regressions:

• U1: Modeled the stage membership at W2.
• U2: Modeled the transition between stages from W2 to W3, conditional on the stage at W2.

No restrictions were imposed on stage membership at W2. However, the action stage was treated as an absorbing state, meaning that participants who reached this stage could not regress to earlier stages. This modeling allowed for six possible transition patterns: (1) static at pre-intention, (2) forward transition from pre-intention to intention, (3) forward transition from pre-intention to action, (4) static at intention, (5) backward transition from intention to pre-intention, and (6) forward transition from intention to action.

#### 2.5.2 Comparison of transition matrices by group

To assess whether the intervention influenced the transitions between stages, two models were compared:

• Model 1: Assumed equal transition matrices across both intervention and control groups.
• Model 2: Allowed for group-specific transition patterns by treating the intervention assignment as a grouping variable.

The fit of the two models was compared using log-likelihood ratio *G*^2^ difference tests, Akaike Information Criterion (AIC), and Bayesian Information Criterion (BIC) (19). The entropy of the models was also examined.

#### 2.5.3 Multinomial logistic regression for transition probabilities

**Figure 2:**
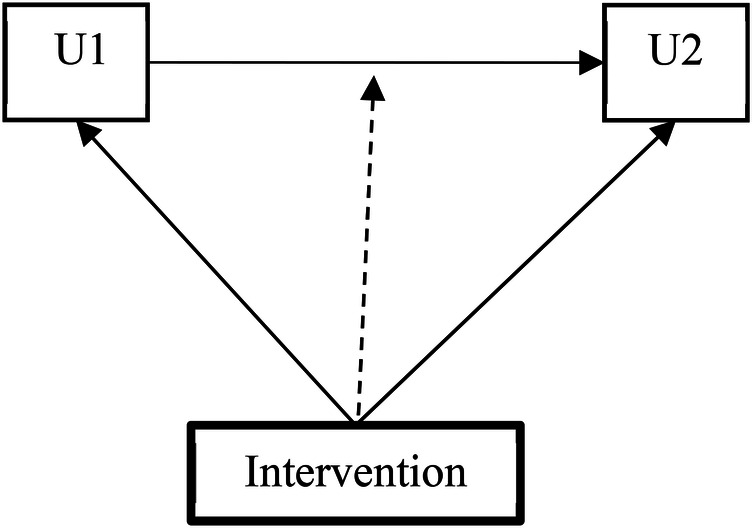
Multigroup Markov model testing the moderating impacts of the intervention on the transition probability.

In Model 1, the intervention assignment was incorporated as a covariate in both the conditional multinomial logistic regression model for U2 (stage transition between W2 and W3) and in the multinomial logistic regression model for U1 (stage membership at W2). In Model 2, a multigroup Markov model estimated group-specific transition matrices, allowing us to test the moderating effect of the intervention on transition probabilities (illustrated in Figure 2).

#### 2.5.4 Intervention efficacy estimates

**Figure 3:**
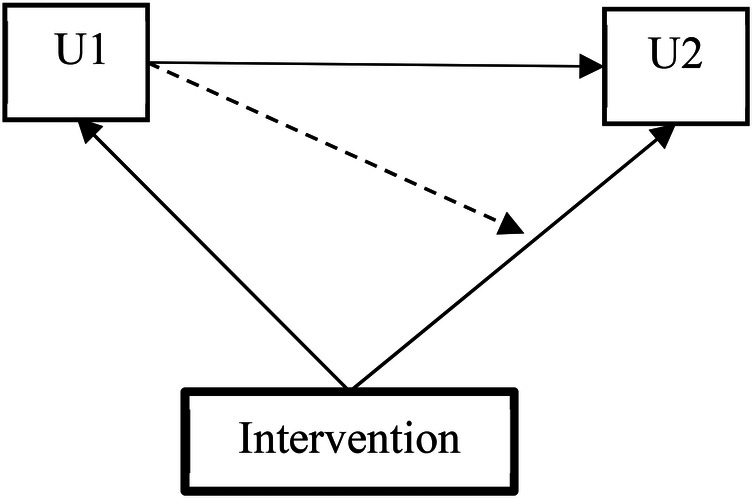
Alternative parameterization of the intervention efficacy.

To further investigate the efficacy of the intervention on transition probabilities, a different parameterization of the model was used (illustrated in Figure 3). Estimates of intervention efficacy on the transition matrix were derived using the conditional multinomial logistic regression presented in Table 1.

**Table 1:** Illustration of the efficacy size of intervention on stage transitions.

Stage transition between W2 and W3 (U2)Stage membership at W2 (U1)	Pre-intention	Intention	Action
Pre-intention	b11+g11*Intervention	b21+g21*Intervention	0
Intention	b12+g12*Intervention	b22+g22*Intervention	0
Action	0	0	0

The b parameters are slopes for the multinomial regression of U2 on U1. The g parameters are slopes for the intervention, varying over the U1 and U2 classes.

#### 2.5.5 Definition of successful and unsuccessful transitions

The efficacy of the intervention was also examined in terms of successful and unsuccessful stage transitions: For pre-intenders, a forward transition to either the intention or action stage was classified as “successful”, whereas no change was deemed “unsuccessful”. For intenders, remaining in the same stage or transitioning forward to action was classified as “successful”, while a backward transition to pre-intenders was deemed “unsuccessful”.

The categorization of “no change” for intenders was justified by the extended time frame (much longer than six months) often required for HIV disclosure. Studies have shown that HIV-positive parents may take years to prepare for disclosure, doing so only when they feel both themselves and their children are ready (20).

#### 2.5.6 Adjustment for baseline covariates

Baseline covariates influencing stage membership at W2 were selected *a priori* (21). Following Streiner's recommendations, ideal covariates should relate to intrinsic characteristics of participants, such as age or sex, or be measured before randomization (22). Moreover, baseline covariates were selected based on their clinical and statistical relevance, as guided by previous observational studies conducted among people living with HIV in Guangxi, China, and prior literature on parental HIV disclosure (23–25).

Parent-level covariates included age, gender, marital status, route of infection, and CD4 count. Child-level covariates included gender and age group (6–9 years, 10–12 years, and 13–15 years). Variance inflation factors (VIF) were calculated to assess multicollinearity among covariates.

#### 2.5.7 Handling missing data

The full information maximum likelihood (FIML) estimator was employed to retain cases with missing data in either wave. FIML provides valid estimates when data are missing completely at random (MCAR) or missing at random (MAR) (26).

#### 2.5.8 Model robustness and convergence

To account for the clustered nature of the data (with participants nested within clinics), a sandwich estimator of standard errors was used. To ensure model convergence on global, rather than local, solutions, random start values were utilized (27).

## 3 Results

### 3.1 Baseline characteristics by intervention group

The baseline characteristics of participants, as summarized in Table 2, indicate that among the 791 individuals included, the average age was 37.7 years. The majority were male (57.5%) and married (76.5%). Approximately 46.5% had completed only primary education, and 46.8% were employed full-time. More than half (54.2%) reported a monthly income of less than 1,000 RMB ($150). Regarding HIV infection, 35.3% were infected by their main partner or spouse, and 23.9% had a CD4 count greater than 500 cells/ml. For children, nearly half (47.7%) were aged 6–9 years, and 53.0% were boys. Randomization checks and attrition analyses revealed no statistically significant differences between the intervention and control groups.

**Table 2:** Baseline socio-demographic characteristics by the intervention and control groups.

Variables	Intervention *N* = 403	Control *N* = 388	Total *N* = 791
Parent level socio-demographics			
Age (SD)	37.6 ± 5.7	37.8 ± 5.4	37.7 ± 5.6
Gender			
Male	231 (57.3%)	224 (57.7%)	455 (57.5%)
Female	172 (42.7%)	164 (42.3%)	336 (42.5%)
Marital status			
Married	303 (75.4%)	301 (77.6%)	604 (76.5%)
Separated/divorced	41 (10.2%)	41 (10.6%)	82 (10.4%)
Widowed	58 (14.4%)	46 (11.9%)	104 (13.2%)
Education completed			
Primary school	169 (43.0%)	186 (50.3%)	355 (46.5%)
Middle school	195 (49.6%)	156 (42.2%)	351 (46.0%)
High school and higher	29 (7.4%)	28 (7.6%)	57 (7.5%)
Employment status			
Unemployed	97 (24.1%)	58 (15.2%)	155 (19.8%)
Part-time	131 (32.6%)	131 (34.3%)	262 (33.4%)
Full-time	174 (43.3%)	193 (50.5%)	367 (46.8%)
Household's monthly income (CNY)			
0–999	20 (57.1%)	199 (51.3%)	429 (54.2%)
1,000–1,999	125 (31.0%)	130 (33.5%)	255 (32.2%)
≥2,000	48 (11.9%)	59 (15.2%)	107 (13.5%)
Clinical-related			
Route of infection			
Spouse/main partner	144 (35.7%)	135 (34.8%)	279 (35.3%)
Commercial sex	121 (30.0%)	143 (36.9%)	264 (33.4%)
Injecting drug use	58 (14.4%)	49 (12.6%)	107 (13.5%)
Others	80 (19.9%)	61 (15.7%)	141 (17.8%)
CD4 group (copies/ml)			
<200	77 (19.5%)	69 (18.8%)	146 (19.1%)
200–349	121 (30.6%)	117 (31.8%)	238 (31.2%)
350–500	99 (25.1%)	98 (26.6%)	197 (25.8%)
≥500	98 (24.8%)	84 (22.8%)	182 (23.9%)
Child-level			
Age group			
6–9	199 (49.3%)	172 (46.0%)	371 (47.7%)
10–12	110 (27.2%)	86 (23.0%)	196 (25.2%)
13–15	95 (23.5%)	116 (31.0%)	211 (27.1%)
Gender			
Male	209 (54.1%)	185 (51.8%)	394 (53.0%)
Female	177 (45.9%)	172 (48.2%)	349 (47.0%)

### 3.2 Descriptive analysis of HIV disclosure stage

The distribution of HIV disclosure stages at W2 and W3 across intervention groups is presented in Table 3. Somers' D statistics indicated a significant difference in disclosure stages between the groups at W3 but not at W2.

**Table 3:** Distribution of HIV disclosure stage at W2 and W3 by intervention group.

Study wave	Intervention group	HIV disclosure stage			Total	Somers’ D
		Pre-intention	Intention	Action		
W2	Intervention	173 (46.8)	133 (36.0)	64 (17.3)	370 (100.0)	0.019, *p* = 0.628
	Control	179 (48.1)	134 (36.0)	59 (15.9)	372 (100.0)	
W3	Intervention	170 (46.1)	127 (34.4)	72 (19.5)	369 (100.0)	0.12, *p* = 0.002
	Control	200 (54.6)	129 (35.3)	37 (10.1)	366 (100.0)	

### 3.3 Intervention specific stage transmission patterns

Model fit statistics presented in Table 4 reveal a statistically significant difference in the *G*^2^ test, indicating that the unrestrained Model 2 was favored over the restrained Model 1 (*p* = 0.021). consequently, unrestrained Model 2 was selected for further analysis. The chi-square test for MCAR showed no statistically significant efficacy (*p* = 0.85), suggesting that the assumption of MCAR was met. The entropy value of 0.95 suggested a good model fit.

**Table 4:** Model fit comparing the restrained model 1 with the unrestrained model 2.

Models	Log-likelihood ratio *G*^2^	H0 value ℓ	Number of free parameters (p)	Degrees of freedom df = W – P - 1	Akaike Information Criterion (AIC)	Bayesian Information Criterion (BIC)
Model 1	5302.09	−2651.04	9	8	5320.09	5361.68
Model 2	5290.58	−2645.29	13	4	5316.58	5376.66
Difference	11.51		4	*p* = 0.021		

Model 1 is a model allowing different group-specific transition patterns. Model 2 is a model with a constraint on equal transition matrix across intervention arms.

### 3.4 Transition probabilities

**Figure 4:**
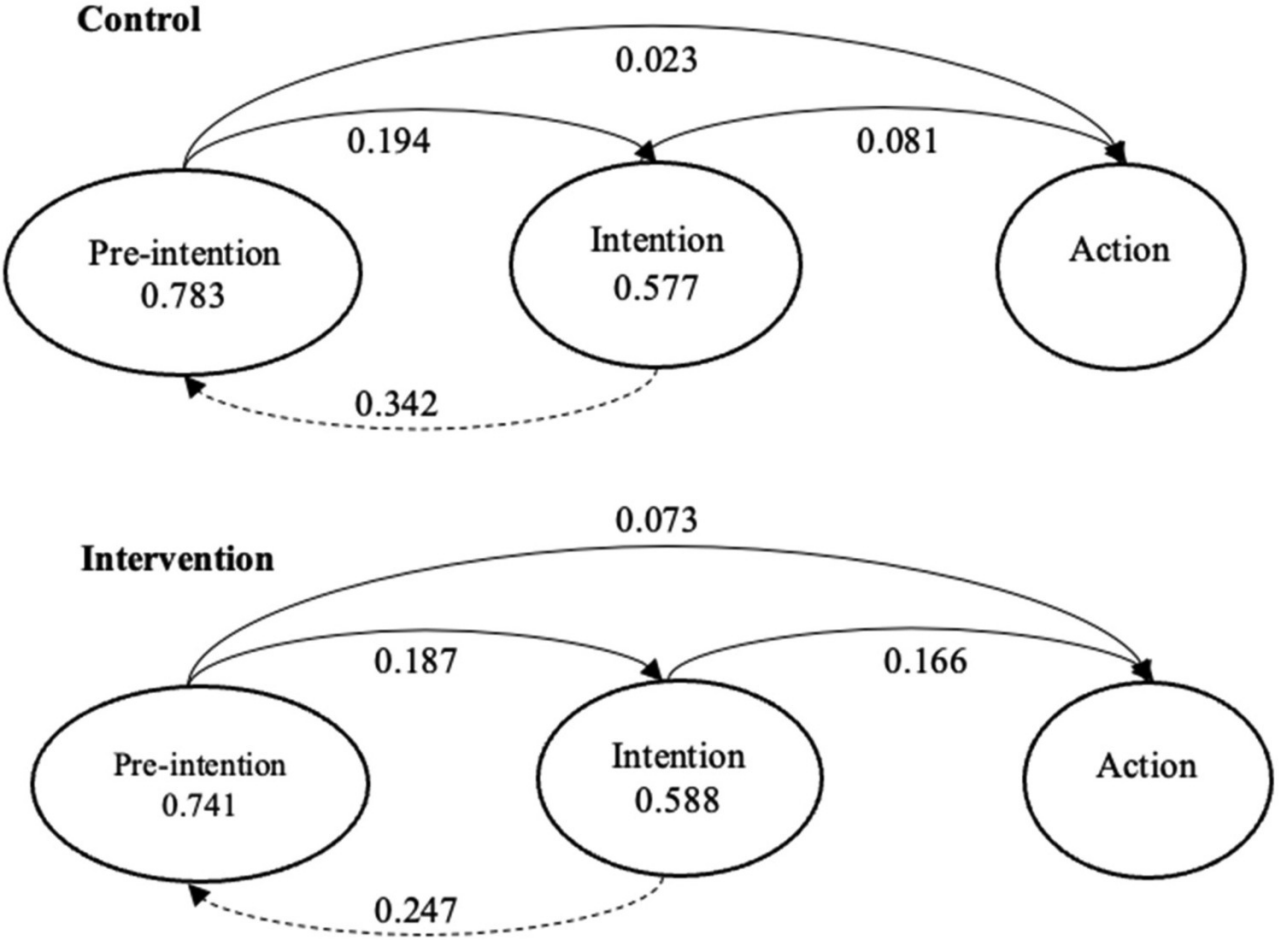
Estimated transition probabilities by intervention groups.

Transition probabilities between HIV disclosure stages are illustrated in Figure 4. The probability of staying in a given stage was represented by a circle and the probability of transition was represented by either solid (for forward movement) or dashed (for backward movement) arrows. In the control group, among pre-intenders at W2, 78.3% stayed static at W3, 19.4% progressed to the intention stage, and only 2.3% progressed to the action stage. Among intenders at W2, 34.2% regressed to the pre-intention stage at W3, 57.7% stayed static, and 8.1% progressed to the action stage. In the intervention group, among pre-intenders at W2, 74.1% stayed static at W3, 18.7% progressed to the intention stage, and only 7.3% progressed to the action stage. Among intenders at W2, 24.7% regressed to the pre-intention stage at W3, 58.8% stayed static, and 16.6% progressed to the action stage.

### 3.5 Intervention efficacy on HIV disclosure stage membership at W2 and stage transition

Intervention efficacy on stage membership at W2 is presented in Table 5. In both the unadjusted and adjusted models, participants in the intervention group had higher odds of being in the pre-intention and intention stages compared to the action stage (reference group), but the differences were not statistically significant. In the adjusted model, the odds ratio (OR) for being in the pre-intention stage was 1.45 (95% CI: 0.66, 3.19; *p* = 0.432), and the OR for being in the intention stage was 1.53 (95% CI: 0.79, 2.97; *p* = 0.289).

**Table 5:** Intervention efficacy on W2 stage membership.

W2 stage membership	Odds ratio	95% CI	*p*-value
Unadjusted model			
Pre-intention	1.37	0.66, 2.84	0.483
Intention	1.36	0.74, 2.48	0.401
Action (ref)			
Adjusted model			
Pre-intention	1.45	0.66, 3.19	0.432
Intention	1.53	0.79, 2.97	0.289
Action (ref)			

Intervention efficacy on stage transitions between Wave 2 and Wave 3 are presented in Table 6. In the unadjusted model, participants in the pre-intention stage at Wave 2 had significantly higher odds of transitioning directly to the action stage (OR = 3.43, 95% CI: 1.17, 10.01), compared to remaining in the pre-intention stage. After adjusting for covariates, this effect became stronger (OR = 6.66, 95% CI: 1.72, 25.8), suggesting that the intervention facilitated movement from pre-intention to action. However, transitions from pre-intention to intention and from intention to action were not statistically significant. Among intenders at Wave 2, the intervention did not significantly influence backward transition to pre-intention (OR = 0.71, 95% CI: 0.35, 1.43) or forward transition to action (OR = 2.01, 95% CI: 0.84, 4.79). Similarly, there was no statistically significant effect on overall successful transition (OR = 1.59, 95% CI: 0.82, 3.09).

**Table 6:** Intervention efficacy on stage transition*.*

W2 stage	W3 stage			Successful transition
	Pre-intention	Intention	Action	
Unadjusted model				
Pre-intention	Ref	1.02 (0.47, 2.20)	3.43 (1.17, 10.01)	1.27 (0.59, 2.71)
Intention	0.71 (0.35, 1.43)	Ref	2.01 (0.84, 4.79)	1.59 (0.82, 3.09)
Adjusted model				
Pre-intention	Ref	1.07 (0.49, 2.35)	6.66 (1.72, 25.8)	1.42 (0.65, 3.13)
Intention	0.69 (0.33, 1.40)	Ref	1.81 (0.74, 4.45)	1.60 (0.80, 3.18)

## 4 Discussion

This study represents one of the first applications of the HAPA to evaluate parental HIV disclosure interventions. Our findings emphasize the theoretical relevance of HAPA in understanding how PLH navigate disclosure processes, demonstrating differential intervention efficacy based on participants' initial stages of disclosure readiness. Specifically, the intervention was more effective among pre-intenders, who were more likely to progress to the action stage rather than remaining static at the pre-intention stage. This finding highlights the potential of targeted interventions to facilitate action-oriented outcomes in PLH who have not yet formed an intention to disclose their HIV status. However, the intervention did not show statistically significant efficacy for those who were already in the intention stage, suggesting that different or additional strategies may be needed to support this group in transitioning to action.

A notable observation in this study is that stage transitions in the disclosure process do not always follow a sequential pattern. Some participants were found to move directly from the pre-intention to the action stage within six months. One possible explanation for this non-sequential transition could be measurement error of stage membership (i.e., misclassification) which has been found to impact the smallest response category (i.e., the pre-intenders) most (28). Another explanation is that the six-month follow-up window was too broad to capture their sequential changes in participants' stages. Therefore, more frequent follow-ups (e.g., every three months) may be necessary in future longitudinal studies to determine whether individuals progress sequentially through the ordered stages within a short time frame or if they tend to skip certain stages.

Moreover, it is possible that participants have disclosed their HIV status without detailed planning and preparation, perhaps due to unexpected circumstances leading to unintended disclosure. This aligns with findings from the transtheoretical transition model (TTM), a widely applied stage-based framework, which suggests that stage transitions may not always be sequential (29, 30). Studies have found that individuals are most likely to skip the preparation stage in TTM and try to move directly from contemplation into action (31, 32). Although the HAPA is conceptualized as a two-stage or three-stage model, limited research has been conducted to examine whether stage skipping is possible in HAPA. Most studies guided by HAPA have posited transition patterns as a sequence from static, regression, to progression without exploring stage skipping (33). Future research should investigate the implications of stage-skipping by comparing transition models that constrain movement between stages with those that allow direct transitions from pre-intention to action. In addition, incorporating qualitative methods, such as in-depth interviews, would provide a deeper understanding of the motivations and circumstances influencing changes in disclosure status, thereby informing the development of more targeted interventions.

The lack of a significant intervention effect on the transition from pre-intention to intention may be attributed to a combination of psychological and social factors. Information deficits, including limited knowledge of the potential benefits of disclosure or uncertainty about how to initiate the conversation, may prevent parents from forming a concrete intention to disclose. In addition, persistent stigma may create hesitation about potential negative consequences, such as discrimination or altered family dynamics, thereby inhibiting progression from pre-intention to intention. Furthermore, parents may not perceive immediate benefits in disclosing their status, especially if they anticipate challenges in their child's emotional processing or fear unintended disclosure by the child to others. Future interventions should incorporate targeted stigma reduction strategies, educational resources that emphasize the advantages of disclosure, and structured decision-making tools to facilitate intention formation and subsequent disclosure planning.

The mixed findings reported in the literature regarding the efficacy of parental HIV disclosure interventions further highlight the complexities of this research area (10). For example, Rotheram-Borus et al. found no statistically significant differences in parental HIV disclosure status between intervention and control conditions over a 24-month period (34). Moreover, previous research has shown that parental HIV disclosure rates are higher among parents with older children, as parents often decide not to disclose to younger children, considering them “too young to understand” (16, 35, 36). Research found that parents of younger children (ages 6–9) demonstrated significant improvements in knowledge, action self-efficacy, and action planning following the intervention, while parents of older children (ages 10–12 and 13–15) showed different patterns of change, including a reduction in perceived benefits of disclosure for the 10–12 age group (16). These results suggest that the efficacy of parental HIV disclosure interventions may vary by child age, with interventions potentially requiring adaptation to address the unique needs of parents depending on the developmental stage of their child.

Our findings suggest that integrating the iCOPE intervention into routine HIV care services could significantly enhance support for parental disclosure. This integration could be accomplished through provider training focused on stage-specific counseling and by incorporating disclosure planning into maternal and child health programs. Notably, the Guangxi CDC has already incorporated our findings into their training plans, manuals, and guidelines for parental HIV disclosure, demonstrating the real-world applicability of our approach within local healthcare systems. However, scaling this intervention will require addressing systemic barriers, including stigma and limitations in healthcare resources. Future research in implementation science is critical to capture the lessons learned from scaling up this intervention, which will be essential for adapting and sustaining the intervention within broader HIV care and maternal-child health programs across various contexts.

Several limitations of this study need to be acknowledged, as they may influence the interpretation of our findings. First, while the intervention and control sessions were conducted with equal intensity, factors such as differential engagement, facilitator variability, or session adherence could introduce bias. Future studies should monitor and address these factors to mitigate potential bias. Second, we assumed no measurement error in categorizing participants' HIV disclosure stages. However, the occurrence of backward stage transitions (i.e., from intention to pre-intention) suggests that measurement error may have been present. To address measurement error, future research should focus on developing and validating more precise stage algorithms that accurately reflect PLH's different stages in the HIV disclosure continuum. Incorporating more complicated latent Markov models that estimates item-response probabilities could also help address this change. Third, due to the limited sample size, we were unable to test whether other stage models with finer stage classifications (e.g., the five-stage TTM) might offer a better fit than the three-stage HAPA in framing HIV disclosure stages. Fourth, due to the small number of participants who reported remaining in the action stage, we were unable to further differentiate these parents based on their disclosure scope (i.e., the specific topics they covered in the disclosure). Lastly, as the question regarding HIV disclosure stage was not asked at baseline, we were unable to depict the full transition profile from baseline to W2, limiting our understanding of early-stage transitions.

Despite these limitations, this study offers significant contribution to the field and highlights important considerations for future research. First, our findings suggest that the three-stage HAPA can be applied to frame and measure HIV disclosure as a process. Second, when assessing the intervention efficacy on stage transitions, considering both specific transition probabilities and overall forward or backward transition patterns in the evaluation enables a more comprehensive investigation of intervention efficacy. Third, the stage-specific intervention efficacy detected in this study indicate that interventions should be tailored to address the unique needs of participants at different stages of behavioral transition. To develop such staged-specific interventions, further research is needed to identify the key predictors of each stage transition, with particular attention to the role of psychosocial factors. Investigating these underlying mechanisms will not only deepen our understanding of the disclosure process but also inform the development of more effective, targeted interventions.

## Data Availability

The data analyzed in this study is subject to the following licenses/restrictions: Due to sensitive information from participants, the data is not publicly available. Requests to access these datasets should be directed to Xiaoming Li, xiaoming@mailbox.sc.edu.

## References

[1] UNAIDS. Global HIV & AIDS statistics — Fact sheet. (2024). Available at: https://www.unaids.org/en/resources/fact-sheet (accessed July 1, 2024).

[2] Li F Feng Y Liu X Hao J Wang D Hu H HBV And HCV co-infection in Chinese newly diagnosed HIV+ subjects in 2015 and 2023: a cross-sectional study. Pathogens. (2024) 13(5):367. 10.3390/pathogens13050367 38787219 PMC11124262

[3] Institute of Social Development Research CCPS. The China Stigma Index Report. (2009).

[4] World Health Organization. Guideline on HIV disclosure counselling for children up to 12 years of age: World Health Organization. (2011). 26158185

[5] Qiao S Li X Stanton B. Disclosure of parental HIV infection to children: a systematic review of global literature. AIDS Behav. (2013) 17(1):369–89. 10.1007/s10461-011-0069-x 22016331 PMC6234003

[6] Qiao S Li X Zhou Y Shen Z Tang Z Stanton B. The role of enacted stigma in parental HIV disclosure among HIV-infected parents in China. AIDS Care. (2015) 27(1):28–35. 10.1080/09540121.2015.1034648 26616123 PMC4685607

[7] Omarzu J. A disclosure decision model: determining how and when individuals will self-disclose. Pers Soc Psychol Rev. (2000) 4(2):174–85. 10.1207/S15327957PSPR0402_05

[8] Chaudoir SR Fisher JD Simoni JM. Understanding HIV disclosure: a review and application of the disclosure processes model. Soc Sci Med. (2011) 72(10):1618–29. 10.1016/j.socscimed.2011.03.028 21514708 PMC4059828

[9] Chi P Li X. Impact of parental HIV/AIDS on children’s psychological well-being: a systematic review of global literature. AIDS Behav. (2013) 17(7):2554–74. 10.1007/s10461-012-0290-2 22972606 PMC3568204

[10] Conserve DF Teti M Shin G Iwelunmor J Handler L Maman S. A systematic review and narrative synthesis of interventions for parental human immunodeficiency virus disclosure. Front Public Health. (2017) 5:187. 10.3389/fpubh.2017.00187 28824896 PMC5545755

[11] Weinstein ND Rothman AJ Sutton SR. Stage theories of health behavior: conceptual and methodological issues. Health Psychol. (1998) 17(3):290. 10.1037/0278-6133.17.3.290 9619480

[12] Schüz B Sniehotta FF Mallach N Wiedemann AU Schwarzer R. Predicting transitions from preintentional, intentional and actional stages of change. Health Educ Res. (2008) 24(1):64–75. 10.1093/her/cym092 18245046

[13] Li X Qiao S Zhou Y. iCOPE, a multi-level, cluster randomized, 36-month, parallel-group study to assess the efficacy of HIV disclosure intervention in HIV parental disclosure among parents living with HIV in China. SAGE Open Med. (2020) 8:2050312120907821. 10.1177/2050312120907821 32128208 PMC7031783

[14] Guangxi CDC. Know your status, embrace health. (2018).

[15] Zhang R Da W Zhou Y Shen Z Qiao S Li X. Psychosocial factors predicting HIV disclosure stage transitions: an evaluation of A theory-based parental HIV disclosure intervention among parents living with HIV in China. AIDS Behav. (2024) 28(1):105–14. 10.1007/s10461-023-04199-6 37812270

[16] Da W Zhang R Zhou Y Shen Z Qiao S Li X. Impact of children’s age on parental HIV disclosure: a parental HIV disclosure intervention among parents living with HIV in China. AIDS Care. (2024) 36(1):76–84. 10.1080/09540121.2024.2308744 38289470 PMC13270385

[17] Stata. Stata 13.0: StataCorp LLC. (2024). Available at: https://www.stata.com/ (accessed May 1, 2024).

[18] Mplus. Mplus 7.4. (2024). Available at: https://www.statmodel.com/ (accessed April 20, 2024).

[19] Collins LM Fidler PL Wugalter SE Long JD. Goodness-of-fit testing for latent class models. Multivariate Behav Res. (1993) 28(3):375–89. 10.1207/s15327906mbr2803_4 26776893

[20] Kennedy DP Cowgill BO Bogart LM Corona R Ryan GW Murphy DA Parents’ disclosure of their HIV infection to their children in the context of the family. AIDS Behav. (2010) 14(5):1095–105. 10.1007/s10461-010-9715-y 20509046 PMC2936671

[21] Agency EM. Guideline on adjustment for baseline covariates in clinical trials. (2015).

[22] Streiner DL. Control or overcontrol for covariates? BMJ Ment Health. (2016) 19(1):4–5. 10.1136/eb-2015-102294 PMC10699339 26755716

[23] Adeoye-Agboola D Evans H Hewson D Pappas Y. Factors influencing HIV disclosure among people living with HIV/AIDS in Nigeria: a systematic review using narrative synthesis and meta-analysis. Public Health. (2016) 136:13–28. 10.1016/j.puhe.2016.02.021 27059370

[24] Hawk ST. Disclosures of maternal HIV infection to seronegative children: a literature review. J Soc Pers Relat. (2007) 24(5):657–73. 10.1177/0265407507081453

[25] Rochat TJ Stein A Cortina-Borja M Tanser F Bland RM. The amagugu intervention for disclosure of maternal HIV to uninfected primary school-aged children in South Africa: a randomised controlled trial. Lancet HIV. (2017) 4(12):e566–e76. 10.1016/S2352-3018(17)30133-9 28843988

[26] Enders CK Bandalos DL. The relative performance of full information maximum likelihood estimation for missing data in structural equation models. Struct Equ Modeling. (2001) 8(3):430–57. 10.1207/S15328007SEM0803_5

[27] Peel D McLachlan GJ. Robust mixture modelling using the t distribution. Stat Comput. (2000) 10:339–48. 10.1023/A:1008981510081

[28] Bassi F Hagenaars JA Croon MA Vermunt JK. Estimating true changes when categorical panel data are affected by uncorrelated and correlated classification errors: an application to unemployment data. Sociol Methods Res. (2000) 29(2):230–68. 10.1177/0049124100029002003

[29] Callaghan RC Herzog TA. The relation between processes-of-change and stage-transition in smoking behavior: a two-year longitudinal test of the transtheoretical model. Addict Behav. (2006) 31(8):1331–45. 10.1016/j.addbeh.2005.10.011 16337342

[30] Kroll C Keller R Scholz U Perren S. Evaluating the decisional balance construct of the transtheoretical model: are two dimensions of pros and cons really enough? Int J Public Health. (2011) 56(1):97–105. 10.1007/s00038-010-0175-y 20697769

[31] Britto C Mehta K Thomas R Shet A. Prevalence and correlates of HIV disclosure among children and adolescents in low-and middle-income countries: a systematic review. J Dev Behav Pediatr. (2016) 37(6):496–505. 10.1097/DBP.0000000000000303 27262128 PMC5949066

[32] Rodkjaer L Sodemann M Ostergaard L Lomborg K. Disclosure decisions: HIV-positive persons coping with disease-related stressors. Qual Health Res. (2011) 21(9):1249–59. 10.1177/1049732311405803 21483024

[33] Zhang C-Q Zhang R Schwarzer R Hagger MS. A meta-analysis of the health action process approach. Health Psychol. (2019) 38(7):623. 10.1037/hea0000728 30973747

[34] Rotheram-Borus MJ Lee MB Gwadz M Draimin B. An intervention for parents with AIDS and their adolescent children. Am J Public Health. (2001) 91(8):1294–302. 10.2105/AJPH.91.8.1294 11499122 PMC1446764

[35] Mugo C Firdawsi O Wang J Njuguna IN Wamalwa DC Slyker JA When they are all grown, I will tell them”: experience and perceptions of parental self-disclosure of HIV status to children in Nairobi, Kenya. BMC Public Health. (2023) 23(1):519. 10.1186/s12889-023-15387-3 36932351 PMC10024367

[36] Murphy DA. HIV-positive mothers’ disclosure of their serostatus to their young children: a review. Clin Child Psychol Psychiatry. (2008) 13(1):105–22. 10.1177/1359104507087464 18411869 PMC2384146

